# Mixture design of α‐pinene, α‐terpineol, and 1,8‐cineole: A multiobjective response followed by chemometric approaches to optimize the antibacterial effect against various bacteria and antioxidant activity

**DOI:** 10.1002/fsn3.3780

**Published:** 2023-11-01

**Authors:** Boutheina Ben Akacha, Monika Michalak, Ivana Generalić Mekinić, Miroslava Kačániová, Moufida Chaari, Faical Brini, Rania Ben Saad, Wissem Mnif, Stefania Garzoli, Anis Ben Hsouna

**Affiliations:** ^1^ Laboratory of Biotechnology and Plant Improvement Centre of Biotechnology of Sfax Sfax Tunisia; ^2^ Collegium Medicum Jan Kochanowski University Kielce Poland; ^3^ Department of Food Technology and Biotechnology, Faculty of Chemistry and Technology University of Split Split Croatia; ^4^ Faculty of Horticulture, Institute of Horticulture Slovak University of Agriculture Nitra Slovakia; ^5^ Laboratory of Microbial Biotechnology and Engineering Enzymes (LMBEE) Center of Biotechnology of Sfax (CBS) University of Sfax Sfax Tunisia; ^6^ Department of Chemistry, College of Sciences at Bisha University of Bisha Bisha Saudi Arabia; ^7^ Department of Chemistry and Technologies of Drug Sapienza University Rome Italy; ^8^ Department of Environmental Sciences and Nutrition, Higher Institute of Applied Sciences and Technology of Mahdia University of Monastir Monastir Tunisia

**Keywords:** mixture design, natural components, pathogenic bacteria, principal component analysis, synergistic effect

## Abstract

α‐Pinene, α‐terpineol, and 1,8‐cineole are compounds naturally present in essential oils, although their amounts vary from oil to oil. Although several studies have reported their antibacterial and antioxidant effects, there are few reports on the synergistic or antagonistic effects of their combinations. The objective of this study was to investigate the combined antibacterial effect of these three compounds. To our knowledge, this is the first report on the prediction of their optimal combination using the mixture design approach. The experimental antibacterial activity of the α‐pinene, α‐terpineol, and 1,8‐cineole mixtures depended on the proportion of each compound in the mixture and the target strain, with minimum inhibitory concentrations (MIC) ranging from 0.31 to 1.85 mg/mL. Using the increased simplex‐centroid mixture design, the mixture containing 0.33% of each molecule proved to be the most effective against *Bacillus cereus* and had the lowest MIC values. In addition, α‐pinene, α‐terpineol, and 1,8‐cineole showed significant antioxidant activity against 2,2‐picryl‐1‐hydrazyl radical (DPPH), with IC_50_ values of 24.53 ± 0.05, 65.63 ± 0.71, and 63.58 ± 0.01 μg/mL, respectively. Statistical planning and the development of utility profiles of the substance mixtures can predict the optimal composition that will exhibit the highest antibacterial activity against *B. cereus* as well as antioxidant properties. Furthermore, the synergistic effect of the mixtures can contribute significantly to their successful use as natural preservatives in various applications.

## Graphical abstract

In this paper, we studied the antibacterial effect against *Bacillus cereus* and antioxidant activity of a mixture of three compounds—α‐pinene, α‐terpineol, and 1,8‐cineole—using chemometric approaches.

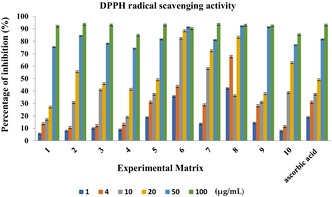

## 1 INTRODUCTION

Microbial foodborne diseases become more aggressive every year, and foodborne pathogens become increasingly resistant (Kim et al., 2019; Msimango et al., 2023; Serwecińska, 2020). Food poisoning and infectious illnesses caused by the consumption of spoiled food are the two main categories of foodborne bacterial diseases (Brandão et al., 2023; Hoffmann & Scallan, 2017). The first is caused by the infectious activity of a live, ingested microorganism, while the second is caused by a microbial toxin present in consumed food (Bari & Yeasmin, 2018). It is typical to distinguish between illnesses and foodborne infections. The toxin‐producing microorganisms are considered pathogenic although they are not infectious; their pathogenicity has only a toxicogenic component (Redmond & Griffith, 2007). The illness is present without the pathogen being ingested. The toxins exert a negative effect on the regular metabolism of the host cells very quickly (a few minutes to a few hours; Jiang et al., 2014). On the other hand, the formation of these metabolites requires systematic development of the producing bacteria in the food, and the conditions for their formation are usually more stringent than the growth conditions (Bonnet et al., 2019; Najafpour, 2007). Some strains of *Bacillus cereus* are responsible for an increasing number of foodborne illnesses in industrialized countries. *Bacillus cereus* is also known to produce an emetic enterotoxin called cereulide. This cyclic peptide is not destroyed by conventional food processing and is not affected by gastric acid or digestive proteases. Cereulide is absorbed into the blood through the digestive tract and distributed throughout the body. In the stomach and small intestine, cereulide can bind to the 5‐HT3 receptor, leading to suppression of mitochondrial activity via the fatty acid oxidation pathway and subsequent activation of afferent vagus nerves, resulting in an induced vomiting mechanism (Agata et al., 1995; Bauer et al., 2018; Decleer et al., 2018; Häggblom et al., 2002; Sergeev et al., 2006). The cytotoxic mechanism of action of cereulide is based on the destruction of the mitochondrial transmembrane potential. K^+^‐ion flows from the outer membrane to the negatively charged inner membrane, disrupting the electrochemical gradient and causing depolarization, followed by swelling of the mitochondria and lack of ATP dynamics. This leads to impaired respiratory function (Jääskeläinen et al., 2003; Mikkola et al., 1999). Many studies have shown that cereulide acts as a membrane‐penetrating ionophore and thus acts as a potassium transporter (Ladeuze et al., 2011; Mikkola et al., 1999). In summary, cereulide forms an ion channel that facilitates diffusion within the cell membrane through the hydrophobic part, maintaining the hydrophilic compartment in the cyclic molecular structure and allowing K^+^ to pass through the membrane. The internal cyclic dodecapsipeptide structure, containing various amino acids and hydroxy acid residues, facilitates the formation of complexes with K^+^, resulting in K^+^ binding and slow release into the mitochondrial matrix (Dommel et al., 2011; Jääskeläinen et al., 2003). After K^+^ dissociation, free cereulide diffuses into the cytosol and prepares for subsequent transport. In this way, continuous K^+^ uptake by mitochondria could continue as long as the membrane potential gradient persists (Jääskeläinen et al., 2003; Teplova et al., 2006). For centuries, synthetic antibiotics have been used to treat foodborne bacterial infections. Due to their abusive use, however, this solution triggers resistance in the pathogens (Ben Hsouna, Michalak, Kukula‐Koch, et al., 2022; Serwecińska, 2020; Thaker & Wright, 2015). The development of antibiotic resistance in foodborne zoonotic bacteria is due to the massive global use of antibiotics in livestock production (Manyi‐Loh et al., 2018). The search for natural antibiotics has become fundamental for human health, and essential oils (EOs) represent an inexhaustible source of countless compounds with biological activity (Ben Akacha et al., 2022; Ben Akacha, Ben Hsouna, et al., 2023; Ben Hsouna, Boye, Akacha, et al., 2022; Ben Hsouna, Hfaiedh, Ben Slima, et al., 2022; Bouteraa et al., 2023). EOs play an important role in plants, acting as antibacterial, antiviral, antifungal, and insecticidal agents and protecting plants from predators and pests (Akacha et al., 2022; Ben Hsouna et al., 2013; Ben Hsouna et al., 2014; Castilho et al., 2012). They are used and valued in perfumes and cosmetic products (creams, soaps, etc.), sanitary products, dentistry, agriculture, and the food industry, as preservatives and additives (Abelan et al., 2022; Akacha et al., 2022). EOs are complex mixtures of 20–60 components at various concentrations, in some cases quite high (from 20% to 70%), while others are only present in trace amounts. The highly concentrated components (terpenes, terpenoids, sesquiterpenes, and aromatic ring compounds) usually play a key role in the antimicrobial and other biological effects of EOs (Bakkali et al., 2008). Some of the important compounds in EOs belong to other classes of compounds, such as hydrocarbons, phenols, alcohols, ethers, aldehydes, and ketones (Dhifi et al., 2016).

α‐Pinene (Salas‐Oropeza et al., 2021) is a molecule of great interest with a wide spectrum of activity (e.g., antibacterial, antifungal, antileishmanial, anti‐inflammatory, antioxidant, and neuroprotective) (Allenspach & Steuer, 2021). 1,8‐Cineole, a cyclic monoterpene ether abundantly occurring in nature, is also highly valued for its biological activity (Farhanghi et al., 2022), such as antioxidant, antibacterial, sedative, anesthetic, and analgesic effects (Davis, 2010). Isomers, such as α‐terpineol, β‐terpineol, γ‐terpineol, δ‐terpineol, and terpinen‐4‐ol, the last of which is the most abundant in nature, are widely used as food flavoring additives; moreover, in recent years, α‐terpineol has been increasingly associated with some biological effects, such as anti‐inflammatory, antioxidant, antiproliferative, and antimicrobial activity. Natural‐origin compounds have multiple advantages. The simultaneous antibacterial and antioxidant action allows for the resolution of numerous issues, including antibiotic resistance and the damaging consequences of oxidative stress (Akacha et al., 2022; Ben Akacha, Ben Hsouna, et al., 2023; Ben Akacha, Garzoli, et al., 2023; Ben Akacha, Michalak, et al., 2023; Ben Hsouna et al., 2023; Ben Hsouna, Hfaiedh, Ben Slima, et al., 2022).

In this context, the present study was conducted to promote the development of effective preservatives for food and cosmetic products, using molecules of natural origin, specifically α‐pinene, α‐terpineol, and 1,8‐cineole, by evaluating their antibacterial and antioxidant effects both individually and in combination. In the latter case, an attempt was made to determine the combination capable of optimally inhibiting *B. cereus* and scavenging DPPH free radicals. For this purpose, the augmented simplex‐centric method was used to construct polynomial models describing the relationship among the antibacterial effect, the antioxidant effect, and the exact proportion of each molecule.

## 2 MATERIALS AND METHODS

### 2.1 Chemicals

α‐Pinene, α‐terpineol, 1,8‐cineole, butylated hydroxytoluene (BHT), thiazolyl blue tetrazolium bromide (MTT), and 2,2‐diphenyl‐1‐picrylhydrazyl (DPPH) were purchased from Sigma‐Aldrich Co. Muller–Hinton agar was purchased from Bio‐Rad. MH agar plates and red–violet bile glucose medium were obtained from Oxoid Ltd.

### 2.2 Antibacterial activity

The antibacterial activity of the samples was determined as minimum inhibitory concentration (MIC) and minimum bactericidal concentration (MBC). The target bacterial strains were of natural origin, including 3 g‐positive bacteria, that is, *S. aureus* ATCC 25923 (*S. aureus*), *Listeria monocytogenes* ATCC 1911 (*L. monocytogenes*), and *B. cereus* ATCC 14579 (*B. cereus*), and 2 g‐negative bacteria: *Salmonella enterica* ATCC 43972 and *Escherichia coli* ATCC 25922 (*E. coli*). These strains were selected to test the antibacterial activity of α‐pinene, α‐terpineol, and 1,8‐cineole according to the method described by Ben Hsouna, Boye, Akacha, et al. (2022). The MIC value is the concentration (in mg/mL) of the compound (Kowalska‐Krochmal & Dudek‐Wicher, 2021) at which the microorganisms show no visible growth after overnight incubation at 37°C. The MIC assay was performed in sterile 96‐well microplates with a final volume of 100 μL per well, containing 10 μL of the tested bacterial suspension with a final inoculum concentration of 10^6^ CFU/mL of each bacterial species. The MIC of each bacterial species was determined after adding an indicator of microbial growth (25 μL of 3‐(4,5‐dimethylthiazol‐2‐yl)‐2,5‐diphenyltetrazolium bromide [MTT] solution, 0.5 mg/mL) to each well, followed by reincubation of the plate at 37°C for 30 min.

To measure the minimum bactericidal concentration (MBC) of the compounds, 10 μL was taken from each well and inoculated into Mueller–Hinton agar (MHA) plates. After incubation for 24 h at 37°C, the number of surviving organisms was counted. The MBC was determined as the lowest concentration of each compound (mg/mL) that killed 99% of the bacteria (Akacha et al., 2022).

The experiments on MICs and MBCs were performed in triplicate, and the results are reported as mean ± standard deviation (SD).

### 2.3 Antioxidant activity

Following the method developed by Ben Hsouna, Hfaiedh, Ben Slima, et al. (2022), activity against free radicals was evaluated by monitoring the percentage of inhibition of the DPPH radical by spectrophotometric measurement at 517 nm, followed by calculation of the concentration that ensures 50% of the radical scavenging activity (the 50% inhibitory concentration, IC_50_). Briefly, an aliquot of 2 mL of 0.1 mM methanolic solution of DPPH solution was added to 2 mL of each test compound at different concentrations (1, 4, 20, 40, and 100 μg/mL). After 20 min in the dark, the change in absorbance (*A*) was measured in relation to the reference without the test sample (control). Free radical scavenging activity was estimated as a percentage of inhibition using the following formula:

$$\mathrm{IP}\;\left(\%\right)=\left(A_{\text{control}}–A_{\text{sample}}/A_{\text{control}}\right)\times 100,$$

where IP is the percentage of inhibition and *A* is the absorbance. The IC_50_ value was expressed in μg/mL of the tested compound. The antiradical activity was dose dependent, and the assay was performed in triplicate.

### 2.4 Optimization of the antibacterial and antioxidant activity of the compounds using the methodology of mixture design

The response studied, which was the antioxidant and antibacterial activity of the three mixed compounds (α‐pinene, α‐terpineol, and 1,8‐cineole), was expressed as the mean inhibitory concentration (IC_50_) for antioxidant activity and as the MIC and MBC for antibacterial activity against *B. cereus*. The optimal response was determined using Minitab 16 software, to determine the optimal ratio of compounds in the mixture. The results are presented in two graphs (mixture contour graph and Cox response trace plot) of the optimal response.

#### 2.4.1 Experimental factors and scope of experiment

**FIGURE 1:**
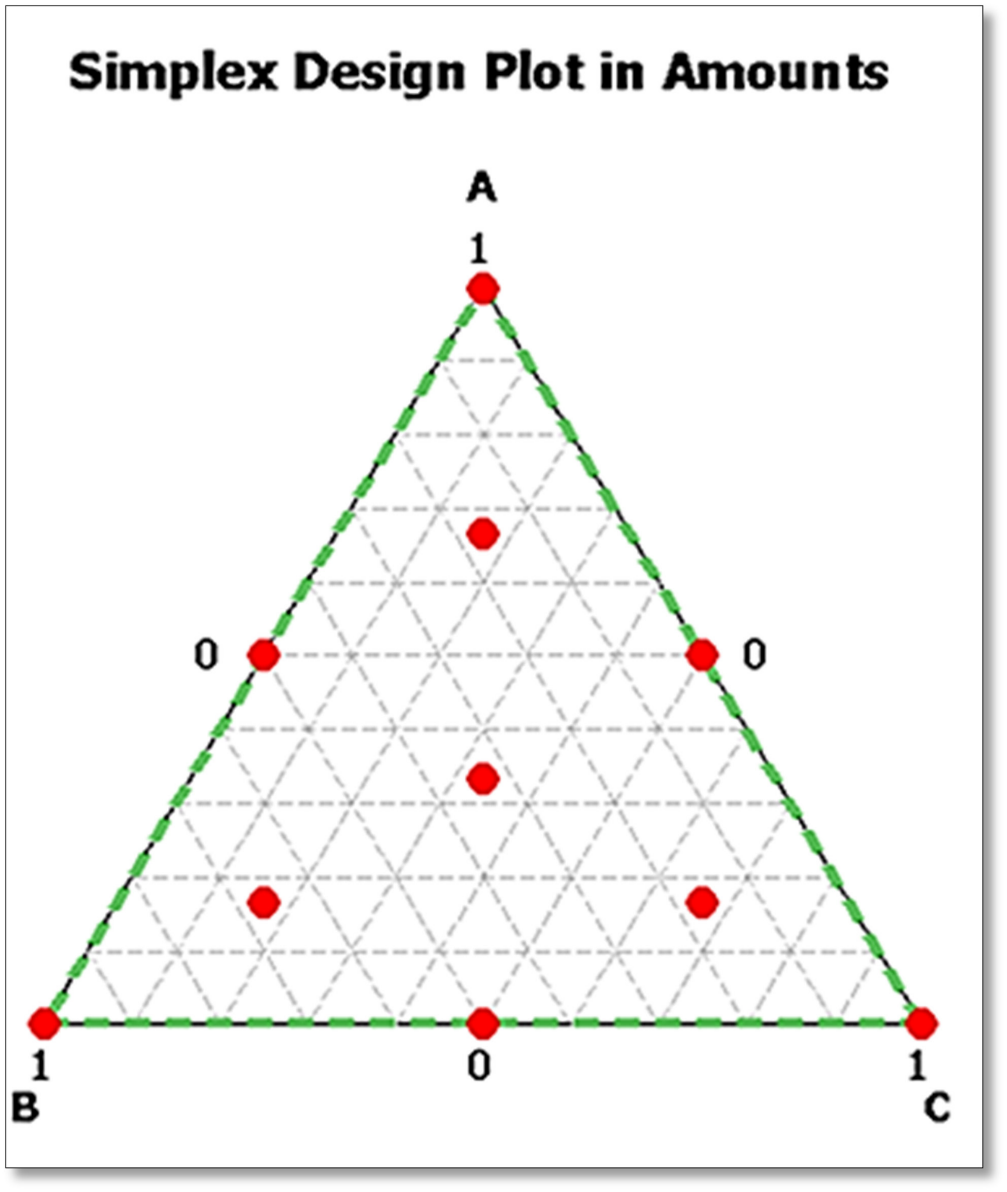
An overview of the augmented simplex‐centroid design for a mixture of the compounds under study.

A modernized simplex‐centroid design was used to evaluate the antioxidant and antibacterial properties of the compounds (Vaillé & Goupy, 2006). The factors are the percentages of each compound in the mixture, varying from 0 to 1, without limiting the design space. The points of the proposed tests are shown in Figure 1. The vertices of the triangle (1, 2, and 3) correspond to the three compounds independently: α‐pinene (**A**), α‐terpineol (**B**), and 1,8‐cineole (**C**). Binary combinations are assigned to the midpoints of the three sides of the triangle, while ternary combinations are assigned to the central point (centroid) and the three augmented points. Table 1 shows the full experimental design for each test, which includes 10 trials, 4 of which are ternary combinations.

**TABLE 1:** Experimental matrix of mixture design of α‐pinene (**A**), α‐terpineol (**B**), and 1,8‐cineole (**C**) with predicted results of their activity (antimicrobial and antioxidant).

Experimental matrix of mixture design				Predicted response		
Runs	Compound ratio in a mixture			MIC (mg/mL)	MBC (mg/mL)	DPPH (IC_50_) (μg/mL)
	α‐pinene (**A**)	α‐terpineol (**B**)	1,8‐cineole (**C**)			
1	1	0	0	0.97	5	25.27
2	0	1	0	1.82	4.94	63.95
3	0	0	1	1.79	4.94	63.79
4	0.5	0.5	0	0.93	3.69	22.16
5	0.5	0	0.5	0.45	3.69	12.21
6	0	0.5	0.5	0.21	1.44	11.10
7	0.3	0.3	0.3	0.20	0.58	0.50
8	0.6	0.1	0.1	0.46	2.61	6.58
9	0.1	0.6	0.1	0.66	1.84	19.10
10	0.1	0.1	0.6	0.49	1.84	15.73

Abbreviations: DPPH, 2,2‐diphenylpicrylhydrazil radical; MBC, minimum bactericidal concentration, MIC, minimum inhibitory concentration.

The experimental matrix (Table 1) was created using 10 mixtures of the following ingredients in different amounts (μg/mL): α‐pinene (**A**), α‐terpineol (**B**), and 1,8‐cineole (**C**). The table also shows the predicted values for MIC, MBC, and IC_50_.

The last step was to fit the data obtained with a special cubic polynomial model by applying least squares regression to estimate the unknown coefficients of the following equation:

$$Y=b_{1}X_{1}+b_{2}X_{2}+b_{3}X_{3}+b_{12}X_{1}X_{2}+b_{13}X_{1}X_{3}+b_{23}X_{2}X_{3}+b_{123}X_{1}X_{2}X_{3},$$

where *Y* is the response, *b*_*i*_ is the magnitude of the effect of each component *X*_*i*_, *b*_*ij*_ is the magnitude of the interactive effect of two components, and *b*_*ijk*_ is the magnitude of the interactive effect of three components on the response. *X*_*i*_ denotes the proportions of component (*i*) in the mixture.

### 2.5 Statistical analysis

Principal component analysis (PCA) was performed using XLSTAT for Windows (v. 2014.1.08; Addinsoft). This method was used to determine the possible relationship between the various mixtures of the compounds and the corresponding biological activities (antioxidant and antibacterial). The type of PCA was Pearson's PCA (*n*), the type of graph was the correlation Biplot, and the coefficient was automatic. All tests were performed in triplicate and expressed as mean ± standard error of the mean (SEM) of the measurements using SPSS software version 26.00 for Windows (SPSS Inc.). Variance was analyzed by one‐way analysis (ANOVA), and Duncan's test was applied to compare each parameter at *p* < .05.

## 3 RESULTS AND DISCUSSION

### 3.1 Antibacterial activity

The MIC and MBC are criteria for measuring the antibacterial performance of compounds (Rodríguez‐Melcón et al., 2022). The MICs and MBCs of α‐pinene, α‐terpineol, and 1,8‐cineole are shown in Tables 2 and 3, respectively. The results showed that all three compounds exhibited significant antibacterial activity against the five bacterial strains. The MICs of α‐pinene (mixture 1), α‐terpineol (mixture 2), and 1,8‐cineole (mixture 3) against *S. aureus* were 1.87 ± 0.62, 1.87 ± 0.62, and 0.45 ± 0.14 mg/mL, respectively, indicating that all tested components had potent bacterial inhibitory activity. The same results were obtained for the other strains, except *E. coli*, for which the MICs were the same for the first three mixtures (0.45 ± 0.14 mg/mL).

**TABLE 2:** Experimental results for minimal inhibitory concentrations (MICs) against different bacteria obtained by testing the mixtures.

MIC (mg/mL)	Gram‐positive			Gram‐negative	
	*Staphylococcus aureus ATCC 25923*	*Listeria monocytogenes ATCC 1911*	*Bacillus cereus ATCC 14579*	*Salmonella enterica ATCC 43972*	*Escherichia coli ATCC 25922*
1	1.87 ± 0.62^b^	1.85 ± 0.65^b^	0.91 ± 0.65^ab^	1.85 ± 0.65^ab^	0.45 ± 0.14^a^
2	1.87 ± 0.62^b^	1.85 ± 0.65^b^	1.85 ± 0.29^b^	1.85 ± 0.65^ab^	0.45 ± 0.14^a^
3	0.45 ± 0.14^ab^	0.45 ± 0.14^ab^	1.85 ± 0.65^b^	3.75 ± 0.29^a^	0.45 ± 0.14^a^
4	0.78 ± 0.07^a^	0.45 ± 0.15^a^	0.91 ± 0.65^ab^	0.45 ± 0.14^a^	0.31 ± 0.05^a^
5	0.22 ± 0.07^a^	0.45 ± 0.14^a^	0.45 ± 0.14^a^	0.91 ± 0.29^a^	0.45 ± 0.14^a^
6	0.22 ± 0.07^a^	0.45 ± 0.14^a^	0.31 ± 0.65^a^	0.91 ± 0.29^a^	0.45 ± 0.14^a^
7	0.46 ± 0.16^ab^	0.45 ± 0.14^ab^	0.25 ± 0.14^a^	0.91 ± 0.29^a^	0.45 ± 0.15^a^
8	1.85 ± 0.65^b^	0.45 ± 0.14^b^	0.45 ± 0.15^ab^	0.91 ± 0.29^a^	1.85 ± 0.65^b^
9	1.87 ± 0.65^b^	0.67 ± 0.52^b^	0.91 ± 0.14^a^	0.91 ± 1.25^b^	1.87 ± 0.65^b^
10	1.85 ± 0.65^b^	0.75 ± 0.44^b^	0.45 ± 0.65^a^	0.91 ± 0.62^ab^	0.91 ± 0.29^ab^

*Note*: The values are expressed as mean ± SEM (*n* = 3). Mixture design (1–10) is given in Table 1.

a,b: Averages with different letters in the same column, for each parameter, are different (*p* < 0.05).

**TABLE 3:** Experimental results for minimum bactericidal concentrations (MBC) against different bacteria obtained by testing the mixtures.

MBC (mg/mL)	Gram‐positive			Gram‐negative	
	*Staphylococcus aureus* ATCC 25923	*Listeria monocytogenes* ATCC 1911	*Bacillus cereus* ATCC 14579	*Salmonella enterica* ATCC 43972	*Escherichia coli* ATCC 25922
1	<5	<5	5 ± 0.00^b^	<5^b^	<5
2	<5	<5	5 ± 0.00^b^	<5^b^	<5
3	3.75 ± 1.25	<5	5 ± 0.00^b^	2.5 ± 1.25^ab^	3.75 ± 1.25
4	3.75 ± 1.25	5 ± 0.00	3.75 ± 1.25^ab^	<5^b^	3.75 ± 1.25
5	<5	5 ± 0.00	3.75 ± 1.25^ab^	<5^b^	<5
6	0.92 ± 0.32	<5	1.56 ± 0.94^ab^	0.9 ± 0.3^ab^	<5
7	<5	<5	0.92 ± 0.94^a^	<5^b^	<5
8	<5	<5	1.56 ± 0.94^a^	3.75 ± 1.25^a^	<5
9	<5	<5	1.56 ± 0.32^a^	1.56 ± 0.94^a^	<5
10	<5	2.5 ± 0.25	1.56 ± 0.94^a^	1.56 ± 0.94^a^	3.75 ± 0.62

*Note*: The values are expressed as mean ± SEM (*n* = 3). Mixture design (1–10) is given in Table 1.

a,b: Averages with different letters in the same column, for each parameter, are different (*p* < 0.05).

These results are consistent with previous reports showing that α‐pinene was effective against several gram‐negative and gram‐positive bacterial strains, including methicillin‐resistant *S. aureus* (IC_50_ = 68.6 ± 7.9 μg/mL) (de Sousa Eduardo et al., 2018). Its antibacterial mechanism involves the enhanced expression of antimicrobial efflux pumps and interactions with metabolic pathways (Kovač et al., 2015).

Some studies have reported that α‐terpineol and terpinen‐4‐ol also showed potent antibacterial effects against gram‐positive bacteria, such as *S. aureus*, *Streptococcus agalactiae* (Johansen et al., 2022), *Bacillus coagulans*, and *Micrococcus luteus* (Kotan et al., 2007), suggesting that α‐terpineol and terpinen‐4‐ol are effective bacterial inhibitors with a broad bacterial spectrum. In the same context, previous publications have shown that 1,8‐cineole has relatively potent antimicrobial activity as a bactericide against many pathogens and spoilage organisms, such as *S. aureus*, *P. aeruginosa*, *E. coli*, and *B. subtilis* (Cai et al., 2021; Şimşek & Duman, 2017), but the antimicrobial mechanisms of 1,8‐cineole are still not completely clear.

The antibacterial interactions between the investigated compounds were observed in the experiments with mixtures 4–10 (Table 2); the proportions of the compounds were carefully chosen to ensure the reliability of the results. The synergistic effect was present in most experiments; in the case of *S. aureus*, the synergistic effect was obtained mainly in mixture 6, with a MIC of 0.22 ± 0.07 mg/mL, as well as for *B. cereus*.

Antagonistic antimicrobial effects were observed, especially against *E. coli*. The results indicate that even in the case of a combination, the concentration of at least one of the compounds is very difficult to optimize, even if there is a synergistic effect. This is consistent with observations from previous studies regarding the inhibition of other bacteria, such as *L. monocytogenes*, *Yersinia enterocolitica*, *B. cereus*, *Salmonella typhimurium*, and *S. aureus*, in which the concentrations required for inhibition were not acceptable to sensory panelists (Kim et al., 1995).

Table 3 shows the results for the MBCs of the matrix components; in most cases, the effect presented is bacteriostatic with an MBC/MIC ratio of <4, except for *S. enterica*, for which the ratio was >4 (for experiments 2, 6, 9, and 10). This is in agreement with the findings of Ben Akacha et al. (2022), who showed that *Lobularia maritima* EO, containing 3.51% α‐pinene and 13.95% of the three enantiomers of terpineol, has bacteriostatic effects against *S. aureus*, *E. coli*, and *L. monocytogenes*. In a work by Kachkoul et al. (2021), the chemical composition and antimicrobial activity of essential oils from *Mentha pulegium* (*MP*EO), *Eucalyptus camaldulensis* (*EC*EO), *Rosmarinus officinalis* (*RO*EO), and their combinations against three bacterial strains such as *Proteus mirabilis*, *Klebsiella pneumoniae*, and *S. aureus*, were investigated. GC–MS analysis showed that *EC*EO was rich in 1,8‐cineole and *p*‐cymene, *MP*EO had a very high content of pulegone, while α‐pinene and camphene were the main components of *RO*EO. After optimizing a mixture of these three oils, their synergistic effect was demonstrated, in fact, the MIC values recorded were lower than those of the oils taken individually. In a similar context to explain the synergistic effect between essential oil terpenes, the results of Zengin and Baysal (2014) demonstrated that combined treatments with α‐terpineol/linalool, α‐terpineol/eucalyptol (1,8‐cineole), and linalool/eucalyptol had a significant effect on the release of cellular components from both Gram‐negative and Gram‐positive bacteria. They also showed that the single eucalyptol had a more pronounced effect than the other terpenes.

#### 3.1.1 The ternary mixture and its antibacterial effect

**FIGURE 2:**
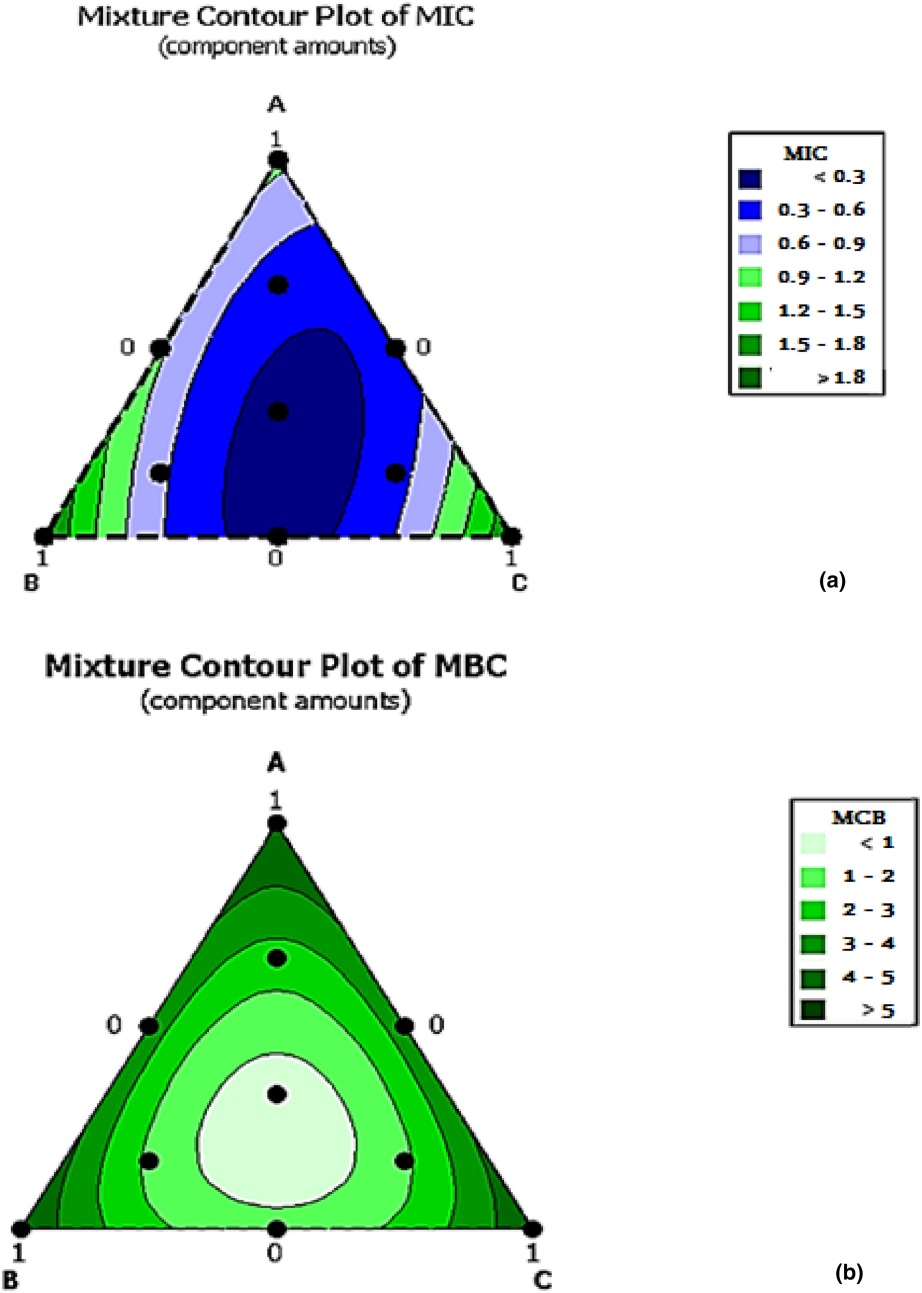
The optimal combination against the bacterial strain shown as a 2D contour plot (a) for minimum inhibitory concentration (MIC) and (b) for minimum bactericidal concentration (MBC).

**FIGURE 3:**
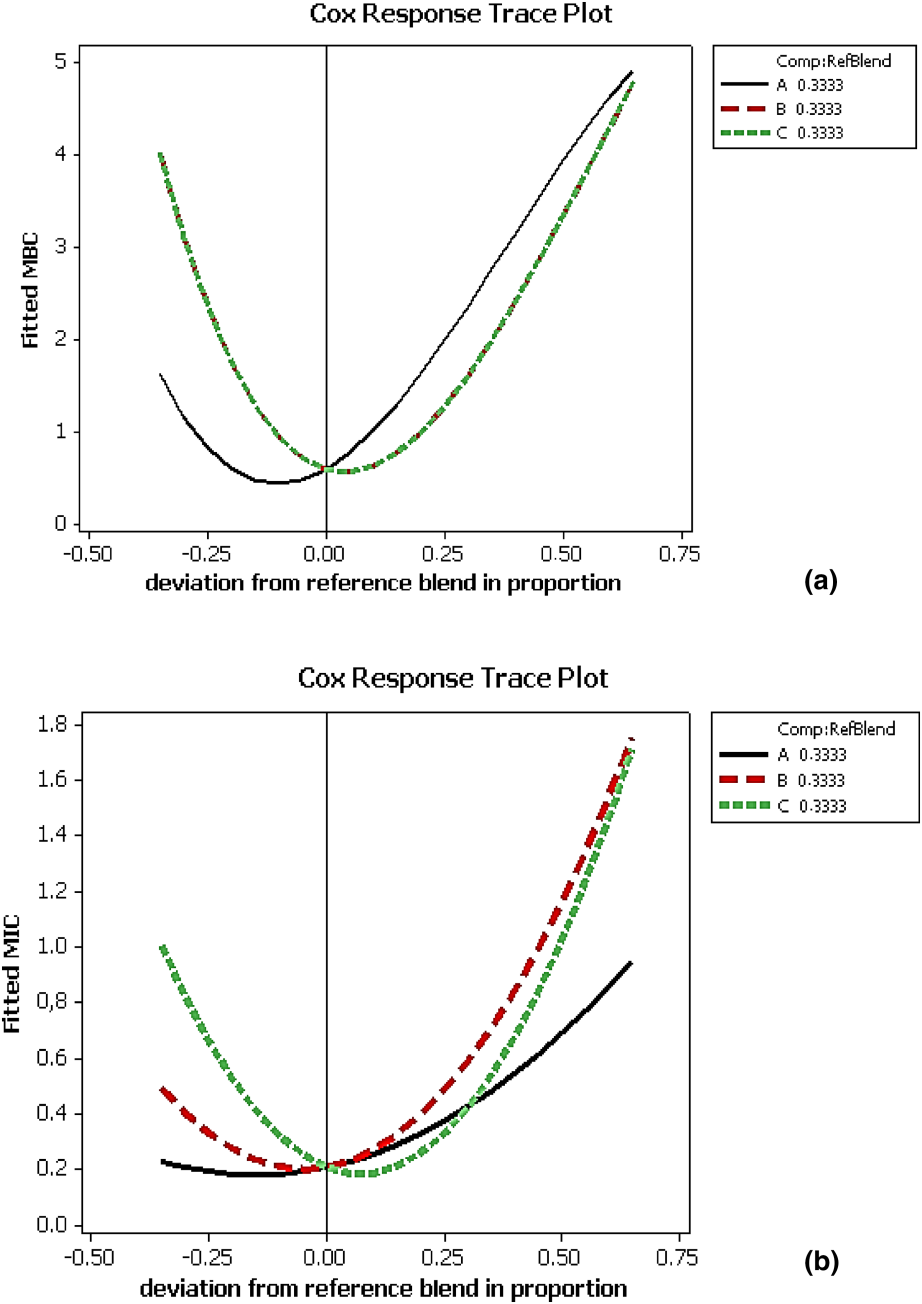
Cox response plots of antibacterial activity against *Bacillus cereus* (a) for minimum inhibitory concentration (MIC) and (b) for minimum bactericidal concentration (MBC).

MICs and MBCs against *B. cereus* ATCC 14579 are presented in Table 1. The bacteria were selected based on the results from Tables 2 and 3 because the strongest synergistic effect was observed with these bacteria and these results represent the maximum MBC values. The ANOVA regression models were established according to the ratio between the three molecules in the mixture (Table 4), and the corresponding responses are shown graphically in Figures 2 and 3. The resulting contours are composed of vertices reflecting the value of the response, and the edges of the triangles represent the concentration of each compound in the binary mixture. The data from the tests were used to analyze the coefficients by regression analysis. ANOVA was used to examine the models for fit and significance, and the significance of the coefficients was determined using *p*‐values.

**TABLE 4:** Results from ANOVA of mixture regression models to optimize the ratios of the compounds for minimum inhibitory concentration (MIC) and minimum bactericidal concentration (MBC) against *Bacillus cereus*.

	DF	Seq. SS	Adj. SS	Adj. MS	*F* value	*p* Value
MIC						
Regression	6	3.12175	3.12175	0.52029	37.97	.006
Linear	2	0.37164	0.67682	0.33841	24.70	.014
Quadratic	3	2.73902	2.08464	0.69488	50.71	.005
AxB	1	0.12139	0.13946	0.13946	10.18	.050
AxC	1	0.63442	0.60578	0.60578	44.21	.007
BxC	1	1.98321	1.76691	1.76691	128.95	.001
Special cubic	1	0.01110	0.01110	0.01110	0.81	.043
AxBxC	1	0.01110	0.01110	0.01110	0.81	.434
Residual error	3	0.04111	0.04111	0.01370	–	–
Total	9	3.16286	–	–	–	–
MBC						
Regression	6	23.5730	23.57302	3.92884	37.63	.006
Linear	2	1.0885	0.00241	0.00120	0.01	.098
Quadratic	3	20.4591	8.82545	2.94182	28.18	.011
AxB	1	3.0637	1.10379	1.10379	10.57	.047
AxC	1	3.1013	1.10379	1.10379	10.57	.047
BxC	1	14.2941	8.26190	8.26190	79.14	.003
Special cubic	1	2.0254	2.02542	2.02542	19.40	.022
AxBxC	1	2.0254	2.02542	2.02542	19.40	.022
Residual error	3	0.3132	0.31318	0.10439	–	–
Total	9	23.8862	–	–	–	–

#### 3.1.2 Effect of the compound mixtures and their interactions

The MIC and MBC results show that the sensitivity of the bacteria to the different mixtures is varied. The best results were obtained for mixture 7, containing equal proportions of α‐pinene, α‐terpineol, and 1,8‐cineole, with an MIC of 0.25 mg/mL, as compared to the other mixtures, with MICs ranging from 0.31 to 0.91 mg/mL. The MBC results correlate with the MICs, with an MBC value of 0.95 mg/mL for mixture 7, which is the lowest value among all the mixtures. However, no antagonistic effect was observed with these combinations. These results are in agreement with a study investigating the antibacterial activity of some terpene compounds from essential oils (α‐terpineol, linalool, 1,8‐cineole, and α‐pinene), both individually and in combination, to determine their interactions at lower doses against pathogenic foods (Zengin & Baysal, 2014). Other studies have shown no antagonistic effect in these mixtures (Ben Hsouna et al., 2013, 2014; Salas‐Oropeza et al., 2021).

For antibacterial activity, linear and cubic *p* values were significant (*p* < .05), and the resulting regression models (Table 4) are shown in the following equations:

(1) $$\begin{array}{cc} \mathrm{MIC}\;B.\;\text{cereus}\;\left(\mathrm{mg}/\mathrm{mL}\right)=\; & 0.978\times X_{1}+1.822\times X_{2}+1.794\times X_{3}\\ & –1.815\times X_{1}X_{2}–3.679\times X_{1}X_{3}\\ & –6.32\times X_{2}X_{3}–0.66\times X_{1}X_{2}X_{3}. \end{array}$$

On the other hand, the MBC results showed that the quadratic and cubic *p* values are significant (*p* < .05), and the regression model is given as follows:

(2) $$\begin{array}{cc} \mathrm{MBC}\;B.\;\text{cereus}\;\left(\mathrm{mg}/\mathrm{mL}\right)= & 5.00\times X_{1}+4.94\times X_{2}+4.94\times X_{3}\\ & –5.11\times X_{1}X_{2}–5.11\times X_{1}X_{3}–13.99\times X_{2}X_{3}\\ & –45.66\times X_{1}X_{2}X_{3}. \end{array}$$

#### 3.1.3 Mixture optimization

Regarding the interaction between the compounds included in the mixture, Figure 2a,b shows that the ternary mixtures have a significant synergistic effect against *B. cereus*. This positive interaction can also be seen in the center of the triangle, which represents the optimal area in the mixture and corresponds to a mixture containing 33.33% of each component. The predicted MIC and MBC values for this point were 0.20 and 0.58 mg/mL, respectively.

As illustrated in Figure 3a,b, the synergistic effect is proportional to the proportion of compounds present in the mixture. The diverse mechanisms of action present in the same combination can explain this result (Selim et al., 2022).

In this context, 1,8‐cineole exhibits different mechanisms of action against bacteria. Antibacterial and anti‐QS activity has been reported against a broad range of pathogenic bacteria by directly inhibiting their biofilm formation as well as reducing the pathogenicity of *E. coli* O101 through blockade of luxS gene expression (Wang et al., 2022). In *Pseudomonas aeruginosa*, 1,8‐cineole significantly inhibited QS‐mediated virulence and biofilm formation (Karuppiah et al., 2021). In other studies, 1,8‐cineole was able to induce protein and nucleic acid leakage in carbapenemase‐producing *K. pneumoniae* cells via an ethidium bromide influx/outflow mechanism. Notably, 1,8‐cineole induced the uptake of ethidium bromide into the cell; this was attributed to the generation of reactive oxygen species which led to lipid peroxidation, thereby damaging the bacterial cell membrane (Moo et al., 2021). In other studies, conducted on *S. aureus*, *E. coli*, and *Moraxella catarrhalis*, pathogens that can cause chronic rhinosinusitis, experiments showed that the pharmacological effect of 1,8‐cineole is not limited to the inhibition of biofilm growth but is an upregulation of the transcripts of the negative regulators A20 and IκB‐α of the NF‐kB pathway was also found, which led to a further attenuation of the inflammatory response (Schürmann et al., 2019). In the same context, Vimal et al. (2017) demonstrated that l‐asparaginase is an antibacterial target of 1,8‐cineole. It should be noted that the mechanisms of action of 1,8‐cineole may vary depending on the type of bacteria against which the compound is active. Furthermore, the effectiveness of this terpenoid may depend on the concentration used and the duration of exposure. 1,8‐Cineole is often used in combination with other antibacterial agents to maximize its effectiveness and reduce the risk of bacterial resistance. The antimicrobial and anti‐inflammatory activities of 1,8‐cineole and its complex mechanisms of action in the regulation of various inflammatory biosynthetic pathways were reviewed by Pries et al. (2023).

On the other hand, α‐terpineol, by influencing the integrity and permeability of cell membranes, can lead to the collapse of the bacterial cell by dissolving the cell wall (Huang et al., 2021). Other studies on the mode of action of α‐terpineol have shown that it can act by blocking DNA, RNA, proteins, polysaccharide synthesis, ATP production, or the tricarboxylic acid cycle (Johansen et al., 2022; Khaleel et al., 2018).

As regards α‐pinene, it is believed that it can alter the fluidity of the membrane or even cross the membrane itself, also leading to cell death. In addition, it increases the expression of antimicrobial efflux pumps and interacts with metabolic pathways (Allenspach & Steuer, 2021; Kovač et al., 2015). The mixture with equal proportions of the compounds proves to be effective against *B. cereus*, and this formulation should be explored as a safe antibiotic.

### 3.2 Antioxidant activity

**FIGURE 4:**
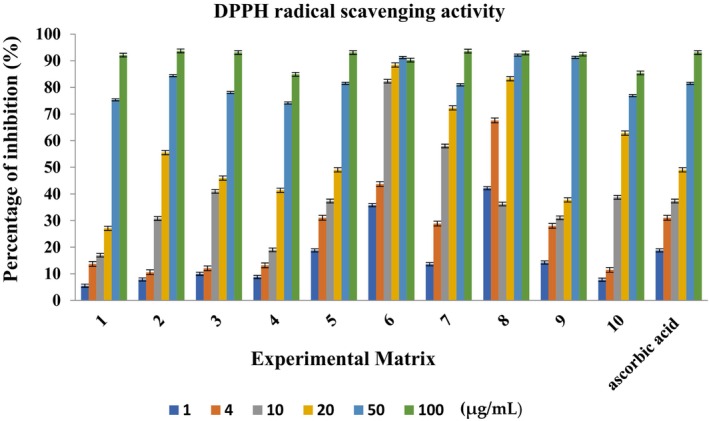
Experimental values of the DPPH inhibition (%) for the various concentrations of the compounds in the mixtures. Mixture designs (1–10) are given in Table 1.

In the DPPH test, the inhibition percentages were 92.15 ± 0.05%, 93.70 ± 0.1%, and 93.10 ± 0.03% for α‐pinene, α‐terpineol, and 1,8‐cineole, respectively, which were determined as free radical scavenging activity (Figure 4). This demonstrates the strong antioxidant capacity of the components, which is in agreement with another study determining the antioxidant and antibacterial activity of seven predominant terpenoids in wine (Wang et al., 2019). That study found that α‐pinene exhibited the highest antioxidant activity in the DPPH assay (Wang et al., 2019).

1,8‐Cineole is an important anti‐inflammatory agent due to the variety of targets on which it can act directly through potent inhibition of LPS‐induced proinflammatory cytokines IL‐1β and IL‐6 (Zuzarte et al., 2022) or by reducing LPS‐induced expulsion and nuclear translocation of the transcription factor Egr‐1 through the extracellular signal‐regulated kinase MEK pathway in the human monocyte cell line THP‐1. Furthermore, in a mouse model of acute lung injury, 1,8‐cineole was shown to suppress NFκB‐dependent expression of MMP9 (matrix metalloproteinase‐9), which belongs to the zinc metalloproteinase family and is involved in the degradation of the extracellular matrix (Khachigian, 2021; Kim et al., 2015). Furthermore, 1,8‐cineole can inhibit bisphenol A‐induced apoptosis and immunosuppression in grass carp hepatocytes by regulating the Wnt/β‐catenin signaling pathway through binding to keap1 (ECH‐like protein 1‐associated Kelch) (Chen et al., 2022). In summary, 1,8‐cineole can act in different ways and the review of the literature, and the most current data underline its complex and holistic mechanisms of action in inflammatory processes. There is extensive evidence that the monoterpenoid 1,8‐cineole has numerous anti‐inflammatory and health‐promoting effects on various human diseases (Zuo et al., 2020). In addition, α‐terpineol exhibits antioxidant activity, as it can suppress superoxide production (Alipour et al., 2022).

The antioxidant activity of the compounds tested in the study reflects their potential to act as preservatives in foods, cosmetics, and pharmaceuticals and to prevent oxidative deterioration of their components.

#### 3.2.1 Predicted and experimental response values (DPPH IC_50_ ) under optimal conditions

Since oxidative stress has been identified as one of the major causes of many diseases, the discovery of new effective antioxidant agents has become essential (Barouki, 2006; Ben Akacha, Michalak, et al., 2023; Ben Hsouna, Michalak, Ben Akacha, et al., 2022). In the present study, the antioxidant activity of different mixtures was determined, and the optimal mixture was detected based on the IC_50_ values of each mixture (Table 5). The IC_50_ values ranged from 65.58 ± 0.71 to 2.65 ± 0.02 μg/mL, while the positive control (ascorbic acid) had a value of 11.26 ± 0.14 μg/mL. The highest antioxidant activity, with an IC_50_ of 2.65 ± 0.02 μg/mL, was observed for the combination of 0.33% each of α‐pinene (**A**), α‐terpineol (**B**), and 1,8‐cineole (**C**) (Table 1).

**TABLE 5:** Response residues of IC_50_: DPPH.

Experimental matrix	Experimental IC_50_ (μg/mL)
1	24.53 ± 0.05
2	65.63 ± 0.71
3	63.58 ± 0.01
4	23.14 ± 0.04
5	11.26 ± 0.64
6	12.57 ± 0.02
7	2.65 ± 0.02
8	8.10 ± 0.14
9	13.36 ± 0.22
10	15.66 ± 0.08

*Note*: The values are expressed as mean ± SEM (*n* = 3). Mixture design (1–10) is given in Table 1.

The fact that this mixture has much stronger activity than its individual components can be attributed to the equal percentages of the main components (Bevilacqua, 2011; Sahraee et al., 2022; Turek & Stintzing, 2013; Zengin & Baysal, 2014), to the synergy between the various components of the oil, or to the macromolecules that act as pro‐oxidants; in this case, the proportions are closer to the natural proportions of these molecules in essential oils (Ben Hsouna, Michalak, Kukula‐Koch, et al., 2022).

The polynomial equation used to determine antioxidant activity has significant *p*‐values for linear and quadratic regression factors (*p* < .05; Table 6).

(3) $$\begin{array}{cc} {\mathrm{IC}}_{50}\;\left(\mathrm{\mu g}/\mathrm{mL}\right)= & 25.3\times X_{1}+64.0\times X_{2}+63.8\times X_{3}–89.8\times X_{1}X_{2}\\ & –129.3\times X_{1}X_{3}–211.1\times X_{2}X_{3}–73.4\times X_{1}X_{2}X_{3}. \end{array}$$

**TABLE 6:** Analysis of variance for IC_50_ using ANOVA.

IC_50_ (μg/mL)	DF	Seq. SS	Adj. SS	Adj. MS	*F* value	*p* Value
Regression	6	4437.76	4437.76	739.63	46.95	.005
Linear	2	761.69	1092.58	546.29	34.67	.008
Quadratic	3	3670.84	2331.01	777	49.32	.005
AxB	1	430.11	340.41	340.41	21.61	.019
AxC	1	888.19	705.62	705.62	44.79	.007
BxC	1	2352.54	1881.31	1881.31	119.41	.002
Special cubic	1	5.23	5.23	5.23	0.33	.605
AxBxC	1	5.23	5.23	5.23	0.33	.605
Residual error	3	47.26	47.26	15.75	–	–
Total	9	4485.02	–	–	–	–

Moreover, the mixture of these three compounds in equal proportions had a significant synergistic effect on *B. cereus*.

**FIGURE 5:**
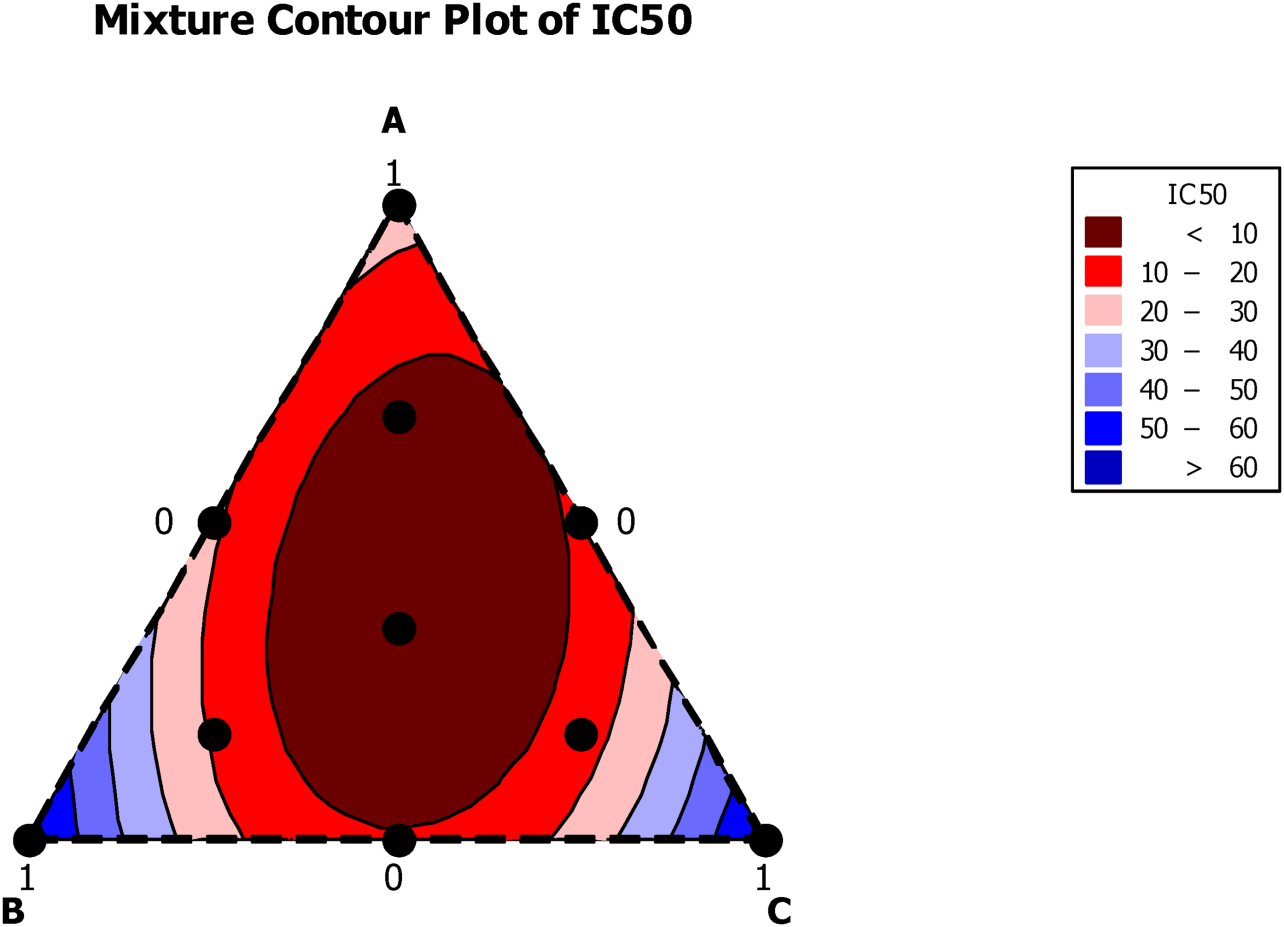
Iso‐response variation of DPPH IC_50_ in mixture design (α‐pinene (**A**), α‐terpineol (**B**), and 1,8‐cineole (**C**).

Regarding the interaction between the three components, these coefficients showed a significant synergistic effect against DPPH free radicals. This positive interaction can also be seen in Figure 5, as the optimal area in the mixing zone is located in the center of the triangle.

**FIGURE 6:**
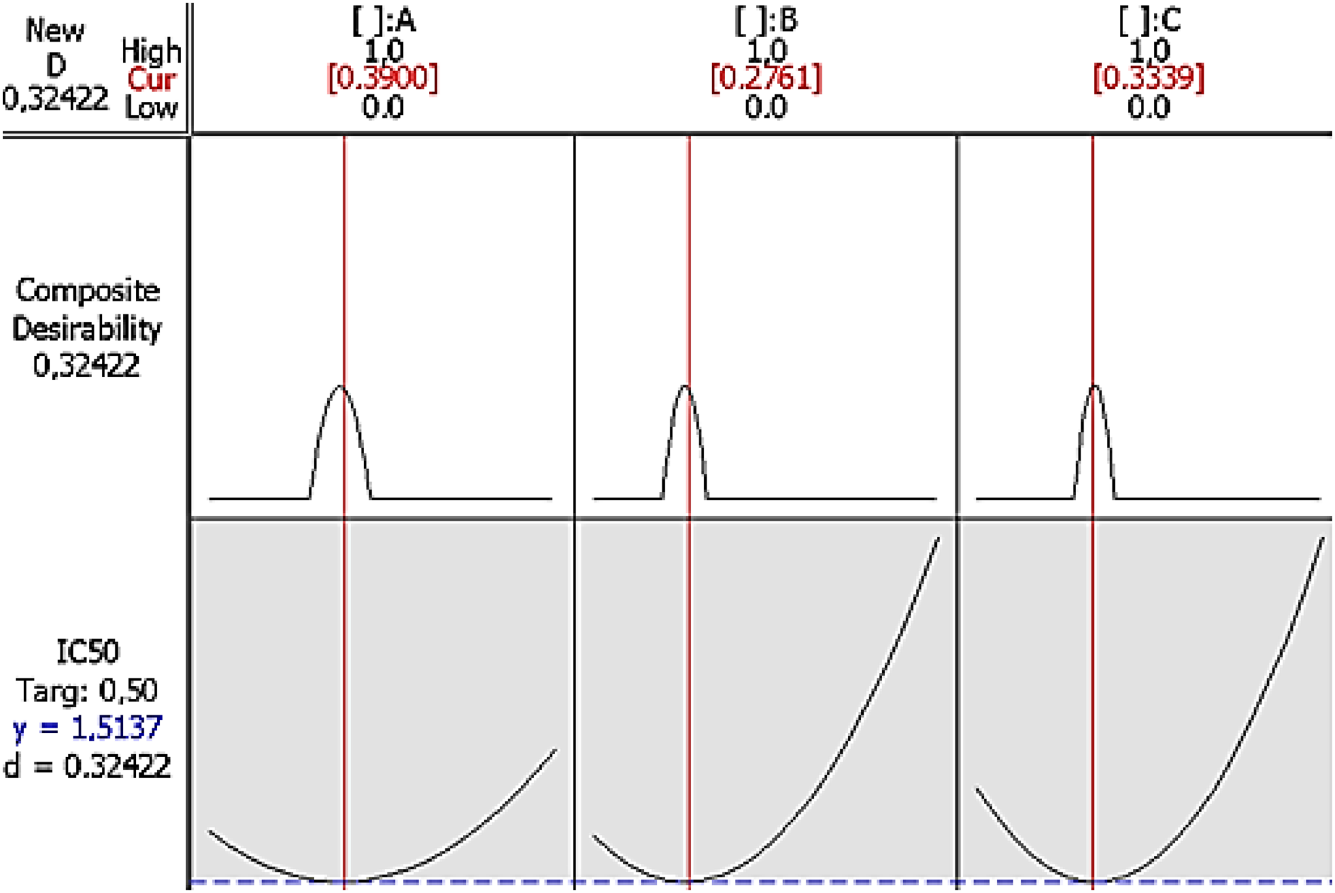
Response optimizer at optimal conditions for minimal IC_50_ response of antioxidant activity (by DPPH method).

To find the most appropriate response, a numerical optimization approach was used for different proportions of the mixture components. The optimal levels were targeted for all responses. This method searches for a set of factor values that simultaneously meet the design requirements for each response. The optimal mixture for the optimal response is the ternary mixture with different proportions. For α‐pinene (**A**), α‐terpineol (**B**), and 1,8‐cineole (**C**), 0.38%, 0.27%, and 0.34%, respectively (Figure 6), were used for DPPH antioxidant activity.

### 3.3 Chemometric and biplot analyses

**FIGURE 7:**
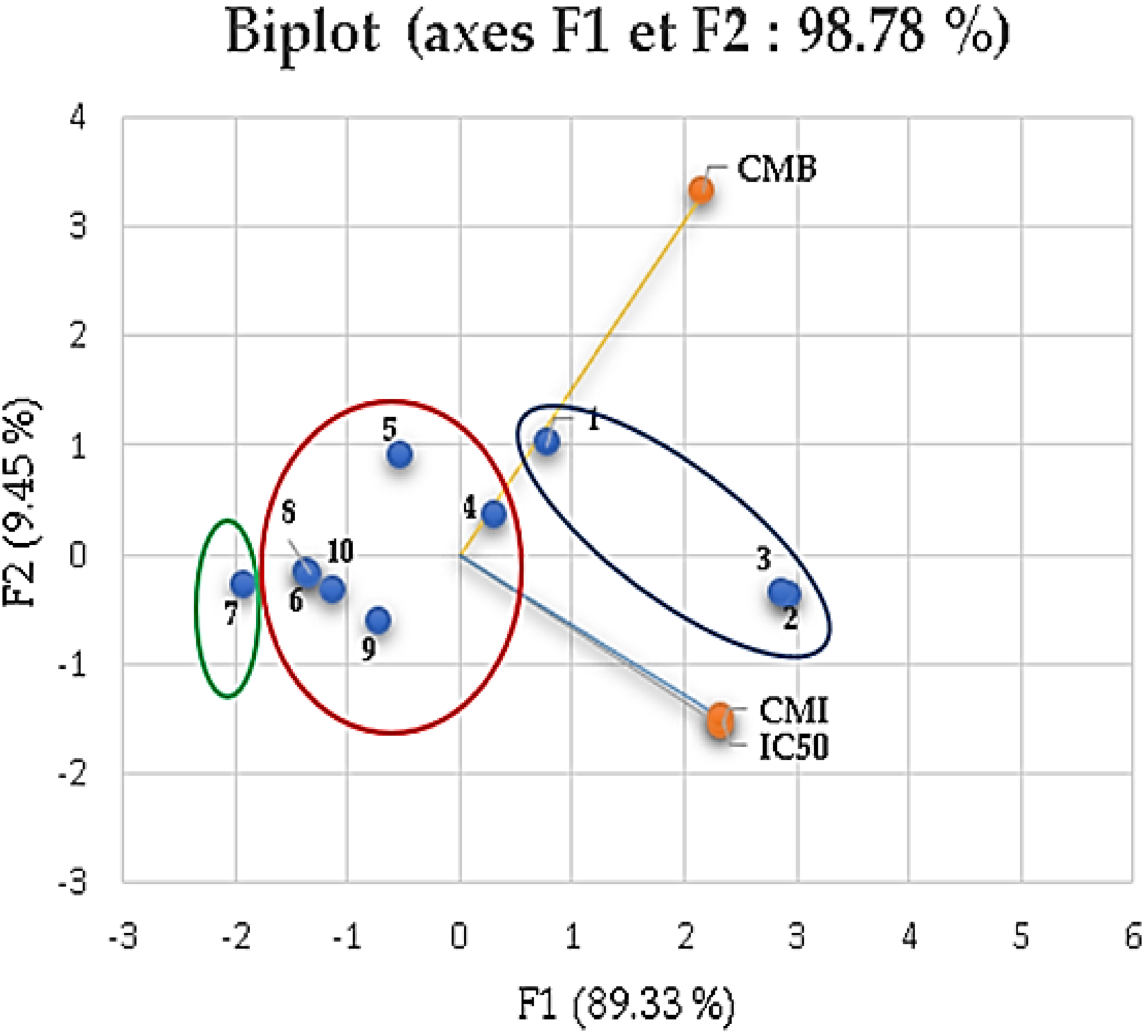
Results of principal component analysis to evaluate the correlation between the results of the optimized and tested mixtures in terms of antibacterial activity against *Bacillus cereus* and free radical scavenging activity (IC_50_).

To verify the results of the mixture design and assess the similarities between the mixtures in terms of MIC, MBC, and CI_50_, the data set was analyzed using chemometric analysis. This includes principal component analysis (PCA) which produces a scoring diagram of the distribution of the various mixtures according to the principal components. In this work, the scoring diagram obtained was presented as a biplot (Figure 7) showing the differences between the mixtures of the three compounds and their bioactivity. The main component is F1, contributing to 89.33% of the total variance, while F2 accounts for 9.45% of the data variance.

Statistical analysis obtained by performing PCA and applying Pearson's correlation indicated that the free radical scavenging activity is positively correlated with antibacterial activity against *B. cereus*, which showed that the lowest antioxidant and antibacterial activities are associated with mixtures 1, 2, and 3 corresponding to the individual effects of α‐pinene, α‐terpineol, and 1,8‐cineole.

Second, a negative correlation was observed among the increases in MIC, MBC, and IC_50_ concentrations and the values obtained by the mixtures. Mixture 7, containing equal proportions of each component, had the highest activity. This is consistent with the results obtained in Sections 3.1.1 and 3.2.1. Mixture 7 represents the optimized mixture with improved efficiency. Some data are available on the interactions between the various compounds of essential oils (Akacha et al., 2022; Alizadeh Behbahani et al., 2017). For example, a study on the antimicrobial activity of the essential oil compounds thymol, carvacrol, linalool, 1,8‐cineole, α‐terpineol, and α‐pinene and their binary mixtures showed effectiveness against bacteria that may be responsible for food spoilage (Bassolé & Juliani, 2012; Zengin & Baysal, 2014).

## 4 CONCLUSION

The biological activities of mixtures consisting of α‐pinene, α‐terpineol, and 1,8‐cineole were improved by using an enhanced simplex‐centroid mixture. In summary, our results show that the tested components, alone or in combination, are effective against *S. aureus*, *L. monocytogenes*, *B. cereus*, *S. enterica*, and *E. coli*. By screening the antimicrobial activity of the three components, we were able to confirm, first, their antibacterial efficacy and the effectiveness of the optimized mixture for the best results. Second, the antioxidant potential of these components was demonstrated using the DPPH assay, and the potential was significantly increased when the three components were combined. According to the mixture model used, the antibacterial and antioxidant efficacy of the studied components depended on the amount of each component in the mixture and on the bacteria targeted. The optimal effect on *B. cereus* and IC_50_ was predicted with a mixture containing 0.33% of each component. By applying the chemometric PCA technique, we can separate all mixtures based on the data and relate the optimal mixture to the best antioxidant and antibacterial activities through correlation models. The results of this study suggest that this mixture could be an alternative natural source of antibacterial agents or preservatives. Furthermore, they should provide an interesting and promising approach to optimize the food preservation process, taking into account both the economic and sensorial aspects of food.

## AUTHOR CONTRIBUTIONS

**Boutheina Ben Akacha:** Conceptualization (equal); data curation (equal); methodology (equal); software (equal); writing – review and editing (equal). **Monika Michalak:** Data curation (equal); software (equal); writing – original draft (equal). **Ivana Generalić Mekinić:** Software (equal); writing – original draft (equal). **Miroslava Kačániová:** Formal analysis (equal); validation (equal). **Moufida Chaari:** Writing – original draft (equal). **Faical Brini:** Funding acquisition (lead); project administration (equal); resources (equal); validation (equal). **Rania Ben Saad:** Data curation (equal); methodology (equal); writing – review and editing (equal). **Wissem Mnif:** Conceptualization (equal); formal analysis (equal); validation (equal); visualization (lead); writing – original draft (equal). **Stefania Garzoli:** Formal analysis (equal); software (equal); writing – original draft (equal); writing – review and editing (equal). **Anis Ben Hsouna:** Conceptualization (equal); data curation (equal); investigation (lead); methodology (equal); project administration (equal); resources (equal); software (equal); supervision (lead); writing – review and editing (equal).

## FUNDING INFORMATION

This research was supported by the Deanship of Scientific Research at University of Bisha‐Saudi Arabia, through the Fast‐Track Research Support Program. This study was supported by a grant from the Ministry of Higher Education and Scientific Research of Tunisia (Program Contract 2022‐2026). The project was financed under the program of the Minister of Education and Science “Regional Initiative of Excellence” in the years 2019–2023, project no 024/RID/2018/19.

## CONFLICT OF INTEREST STATEMENT

The authors declare no conflict of interest.

## Data Availability

No data are available for the Data Availability Statement.
